# Non-adjacent dependency processing (or lack thereof) in bonobos: an artificial grammar experiment

**DOI:** 10.1098/rsos.242173

**Published:** 2025-04-23

**Authors:** Maël Leroux, Nicole J. Lahiff, Chiara Zulberti, Amanda Epping, Calle Uerling, Jared P. Taglialatela, Jutta L. Mueller, Stuart K. Watson, Simon W. Townsend

**Affiliations:** ^1^ Univ Rennes, Normandie Univ, CNRS, EthoS (Ethologie animale et humaine) – UMR 6552, Rennes, France; ^2^ Center for the Interdisciplinary Study of Language Evolution (ISLE), University of Zürich, Zürich, Switzerland; ^3^ Department of Evolutionary Anthropology, University of Zürich, Zürich, Switzerland; ^4^ Institute of Biology, University of Leipzig, Leipzig, Germany; ^5^ Ape Initiative, Des Moines, IA, USA; ^6^ Department of Ecology, Evolution, and Organismal Biology, Kennesaw State University, Kennesaw, GA, USA; ^7^ Institute of Cognitive Science, University of Osnabrück, Osnabrück, Germany; ^8^ Department of Linguistics, University of Vienna, Vienna, Austria; ^9^ Department of Evolutionary Biology and Environmental Studies, University of Zürich, Zürich, Switzerland

**Keywords:** *Pan paniscus*, evolution of language, dependency processing, communication

## Abstract

A key feature of language is our capacity to process syntactic relationships between words, whether they are directly sequential (‘adjacent dependencies’) or separated by other words (‘non-adjacent dependencies’). Recent data suggest that the basic ability to compute adjacent and non-adjacent dependencies is not uniquely human, but rooted deep within our primate lineage, perhaps as far back as our last shared ancestor with chimpanzees and common marmosets (approx. 40 Ma). However, this conclusion hinges on comparable data from other non-human primate species, in particular from bonobos, to whom we are equidistantly related to chimpanzees. To further explore this ancestral hypothesis, we tested if bonobos process both adjacent and non-adjacent dependencies in an artificial grammar learning paradigm. We habituated subjects to strings of arbitrary acoustic stimuli comprised of predictive ‘rules’ between elements that were consistent with adjacent and non-adjacent dependencies. We then tested whether the bonobos were able to (i) apply these rules to novel acoustic stimuli and (ii) detect rule violations. Ultimately, we found no evidence that bonobos processed adjacent or non-adjacent dependencies. This finding ostensibly complicates claims for homologous origins for this capacity, but additional data from other bonobo populations and other great ape species are necessary to draw firm evolutionary inferences.

## 1. Introduction

The structural complexity of human language has been argued to distinguish humans (*Homo sapiens*) from all other animal species [1]. One dimension of language that makes it particularly powerful relative to other communication systems is our capacity for syntax—i.e. the ability to combine words into phrases [2] and thereby generate new meanings [3]. A key feature underlying syntax is our capacity to relate words or phrases with one another, whether they are directly sequential (i.e. ‘adjacent dependencies’, hereafter ‘ADs’) or separated by other elements (i.e. ‘non-adjacent dependencies’ or hereafter ‘Non-ADs’). For example, in the sentence ‘The fox jumped quickly over the lazy dog’, we can readily process the relation between the fox and the dog, despite being separated by a number of other meaningful elements. This massively expands the range of meaningful structures we can create using a limited repertoire of words [4].

Given the critical importance of Non-AD processing for language, recent comparative research has attempted to retrace the evolutionary origins of this capacity by investigating the ability of non-human primates to process Non-ADs. Artificial grammar experiments, where subjects must compute predictive relationships between elements in strings of stimuli organized according to grammatical rules, are a powerful means of probing Non-AD processing capacities in non-human species [5,6]. For example, Guinea baboons (*Papio papio*), who were tested on a serial reaction time task, were able to detect Non-ADs in the visual modality [7]. Furthermore, rhesus macaques (*Macaca mulatta*) were able to detect Non-ADs in the auditory modality when using non-adjacent ordering relationships within sequences of syllable triplets [8]. Until recently, a constraint on this research programme was that these experimental designs were not standardized across species, problematizing direct cross-species comparisons and, consequently, our ability to draw robust evolutionary inferences. However, in the last few years, a standardized artificial grammar procedure to test Non-AD processing in non-human animals has been developed and implemented in marmosets (*Callithrix jacchus*), chimpanzees (*Pan troglodytes*), human adults [9] and even birds (chestnut crowned babblers, *Pomatostomus rucifeps*) [10]. These experiments found that humans, apes and monkeys each processed both ADs and Non-ADs in the auditory domain [9]. Together, these data suggest that the cognitive ability to process ADs and Non-ADs is phylogenetically ancient, rooted at least as far back as our last common ancestor with chimpanzees (approx. 6 Ma), and potentially as far back as our last ancestor with common marmosets (approx. 40 Ma). Moreover, these ancestral origins appear to be reflected in human ontogeny, as newborn infants were also able to process the same artificial grammars [11]. However, it is also possible that the capacity to process Non-ADs evolved convergently in these non-human species, as it has done in some avian species [12]. In order to robustly determine whether Non-AD processing was already present in our last common ancestor with chimpanzees, it is necessary to examine the species with whom we are equidistantly related—bonobos (*Pan paniscus*).

**Figure 1.**
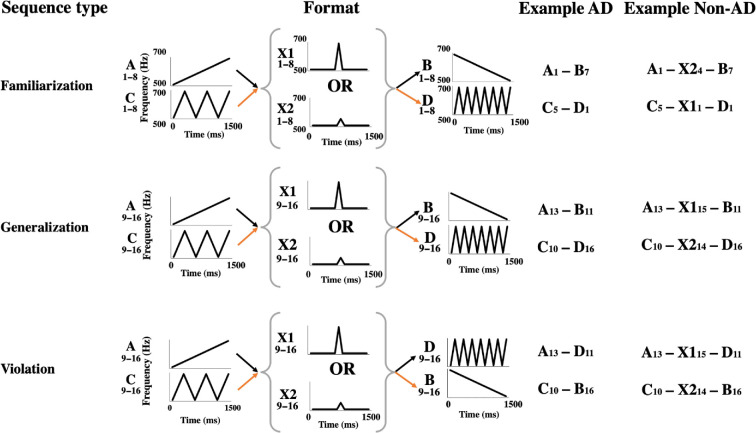
Visual representation of each element and possible transitions between them for each sequence type (bracketed elements apply only to Non-AD condition), with examples. Numbers below the category label indicate possible pitch variants. *Y*-axis values in the familiarization row refer to pitch variant 1 of each element. The arrow colour represents sequence transitions corresponding to a grammar (A–X–B and C–X–D).

Here, we address this by replicating the artificial grammar experiments conducted by Watson *et al*. [9] with bonobos, allowing direct comparisons with findings from chimpanzees and common marmosets. Specifically, we habituated seven captive bonobos to auditory artificial grammars composed of computer-generated sine tones following AD or Non-AD grammars. Our grammars were combinations of two (AD grammar) or three (Non-AD grammar) acoustic elements from six categories of sound types (*A*, *B*, *C*, *D*, *X1*, *X2*; see figure 1). As such, for the AD grammars, individuals were habituated to ‘*A–B’* and ‘*C–D’* sequences, while for Non-AD grammars they were habituated to ‘*A–Xi–B’* and ‘*C–Xi–D’* (where ‘Xi’ could be category X1 or X2) sequences. Once habituated, we tested our subjects using three treatments: familiar sequences, generalization sequences and violation sequences. Familiar sequences were identical to sequences played during habituation. Generalization sequences were structurally identical to familiar sequences but comprised of novel pitch variants of the acoustic elements. Finally, violation sequences were also comprised of novel pitch variants but did not follow the habituated dependencies: for example, ‘*A–D’* represents a violation of the habituated ‘A–B’ grammar (see figure 1).

To measure our subjects’ reaction to these three treatments, we measured the latency until their first look towards the loudspeaker, and the total duration of their gaze towards the loudspeaker during a 15 s window beginning from the onset of the final sound in the sequence. If the capacity to detect Non-ADs is phylogenetically rooted in at least the last common ancestor of *Pan*, we expected bonobos to demonstrate a capacity to process both ADs and Non-ADs, as found in humans and chimpanzees [9]. Specifically, in line with Watson *et al*. [9], we predicted that if individuals processed the dependencies within the grammars, we should find that familiar sequences elicit a very weak or no response (because they have been habituated to them), generalization sequences should elicit either weak or no response (because they are familiar structures but comprised of novel sound variants), and violation sequences should elicit the strongest response (being comprised of both novel sequences and structures). This pattern of results would be reflected by the response measure of gaze duration following the pattern ‘Familiar sequence ≤ Generalization sequence < Violation sequence’, while latency to looking at the loudspeaker would follow the pattern ‘Familiar sequence ≥ Generalization sequence > Violation sequence’.

## 2. Methods

### 2.1. Subjects and study site

**Figure 2.**
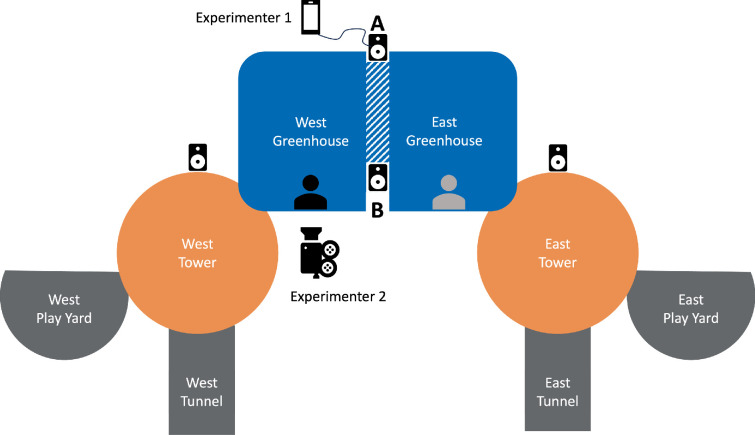
ACCI facility map. In blue: the greenhouse, divided into two sub-areas: the west and east side. In orange: the towers, one on the west and one on the east side. In grey: the outdoor enclosures, a play yard and a tunnel on both the west and the east side. The loudspeaker icons indicate the position of the loudspeakers during the familiarization phase (in the greenhouse, the loudspeaker is in position A for the period of March–April and in position B from October to November). For the testing phase, only loudspeaker A (March–April) or B (October–November) was present. Experimenter 1 represents the position of the first experimenter playing the stimuli, out of sight of the subject. Experimenter 2 represents the position of the second experimenter, filming the subject. The subject is in black while the accompanying individual is represented in grey.

Our subjects were 7 adult bonobos (3 females, 4 males, mean age = 26 years) housed at the Ape Cognition and Conservation Initiative (ACCI), Des Moines, IA, USA (see electronic supplementary material, table S1, for individual demographics) and included the language-trained bonobo Kanzi and other semi-trained individuals [13]. During the study period (March–April and October–November 2022), individuals were housed in groups, the composition of which changed daily in accordance with the fission–fusion social dynamics observed in wild bonobos [14]. Each group had access to both indoor and outdoor enclosures. Indoor enclosures included two ‘towers’ (48 m^2^ each, figure 2) and a greenhouse (125 m^2^, divisible in two equal spaces, figure 2) interconnected via tunnels and shift doors. Outdoor enclosures included two play yards (37 m^2^ each, figure 2) and outside corridors connecting to a larger outdoor area (16 187 m^2^, not available during the study period due to renovation work).

### 2.2. Experimental procedure

In this study, we replicated the artificial grammar experiments conducted by Watson *et al*. [9] on chimpanzees, marmosets and humans. We ran two experimental conditions: the first tested bonobos’ ability to detect adjacent dependencies (ADs, March–April 2022) while the second tested their ability to detect non-adjacent dependencies (Non-ADs, October–November 2022). Both conditions followed the same overall procedure, differing only in the stimuli played back to the subjects. Because the familiarization phase could only be carried out in a group context, we could not counterbalance the presentation of AD and Non-AD conditions between individuals. Therefore, all individuals were first exposed to the AD condition and later the Non-AD condition. A key difference with Watson *et al*. [9] was that, due to the limited available sample size, we ran both AD and Non-AD conditions with the same individuals—leaving seven months between test periods to mitigate risks of habituation to the test stimuli.

### 2.3. Stimuli

Our playback stimuli were identical to the ones described in Watson *et al*. [9] and were strings of computer-generated, frequency-modulated sine tones from six acoustic categories (‘*A’*, ‘*B’*, ‘*C’*, ‘*D’*, ‘*X1’* and ‘*X2’*), generated in 16 pitch-shifted variants (1–8: familiar sequences; 9–16: generalization and violation sequences; see figure 1 and https://osf.io/4m3gv/ [9] for scripts used to generate these acoustic elements in Praat). Each category variant was separated by 50 Hz, starting from 500 Hz (at onset). An exception was the frequency difference between variants 8 and 9 of each category, which were separated by a 200 Hz gap to increase the perceptual difference between ranges 1–8 and 9–16 [9]. All elements had a duration of 1500 ms, with a 10 ms volume fade in/out to eliminate sound onset effects. All elements were generated using Praat [15]. For all sequences, there was a 500 ms gap between elements. AD grammars were composed of one element from each of two dependent categories (‘*A’* & ‘*B’* or ‘*C’* & ‘*D’*), and Non-AD grammars were identical but with the addition of a central ‘*X*’ element (from category ‘*X1’* or ‘*X2’*), separating the two dependent elements (figure 1). Therefore, including gaps between sounds, all AD sequences lasted a total of 3500 ms, and Non-AD sequences lasted 5500 ms. Unlike Watson *et al*. [9], we did not have sufficient available sample size to control for the fact that certain combinations of elements may be more easily learnable than others (e.g. dependencies between A & B versus A & D) by counterbalancing different grammars across groups. Therefore, for our grammars we selected the combination of elements that had a theoretical basis for being the most easily learnable due to the higher similarity between dependent elements [16].

### 2.4. Protocol

#### 2.4.1. Familiarization phase

##### 2.4.1.1. Playback procedure

All subjects were provided with at least 10 familiarization sessions. During these sessions, they were played back 240 familiar sequences presented in a random order with a 2500 ms gap between sequences (120 ‘*A–B’* and 120 ‘*C–D’* for the adjacent dependency condition and 120 ‘*A–X–B’* and 120 ‘*C–X–D’* for the non-adjacent dependency condition). Familiarization sessions were carried out twice per day for 5 consecutive days. To ensure familiarization was optimal independently of the date and time a subject was tested, we ran a familiarization session every morning following these 5 days before testing any individuals on a given day, until all tests were completed.

##### 2.4.1.2. Logistics

To ensure all individuals heard the familiarization equally and therefore had the same level of habituation, all familiarization sessions were carried out indoors, with loudspeakers placed in the greenhouse and towers (see figure 2). Volume levels were controlled such that the sequences were clearly audible from anywhere within the enclosure. Finally, to ensure individuals were habituated to the experimenters and therefore not simply reacting to their presence during testing, both experimenters were present and visible at all times during all familiarization sessions, and regularly interacted with the bonobos outside of these sessions.

### 2.4.2. Test phase

#### 2.4.2.1. Playback procedure

Immediately before test sessions, subjects were exposed to 60 familiar sequences (30 of each grammar) to refamiliarize them with the grammars. After this ‘refamiliarization phase’, the experimenter waited at least 2 min before beginning the test phase. Test sessions were composed of 12 trials in total, with 4 of each stimulus type (familiar, generalization and violation in a randomized order). Trials were marked as invalid if (i) the subject left the camera frame within the 15 s response window set for each trial, (ii) an external noise distracted the subject or (iii) subjects were looking in the direction of the loudspeaker at the onset of the final sound in a sequence. Subjects were only included in the analysis if they had at least one valid trial for each stimulus type (individual ‘EL’ was excluded from further analysis in the Non-AD condition, as she did not meet this criterion).

#### 2.4.2.2. Logistics

The test phase occurred in the greenhouse. Bonobos were encouraged to move into this area either alone or in pairs (if social isolation was deemed likely to cause stress), and the door was closed behind them to prevent interruption from groupmates. When in a pair, the subject was naive while the accompanying individual had already been tested. To limit the influence of a social partner during the test phase, we placed the subject in one part of the greenhouse and the accompanying individual in the other part, separated by a shift door but visible to one another (see figure 2). Subjects were provided with a bottle of diluted juice attached to the mesh of their enclosure at a height easily reached by a seated bonobo. This ensured that subjects remained in-frame of the camera while encouraging them to orient their heads directly forward so that they were reliably looking away from the loudspeaker before a trial was started. In the AD condition, we placed the loudspeaker approximately 150° from where the subject was located (see figure 2, position A). Because of the low rate of response in the AD condition, in the Non-AD condition we reduced the angle at which the loudspeaker was placed from the subject (90°; see figure 2, position B) to circumvent potential motivational issues confounding our results (i.e. the subject was not sufficiently motivated to engage in substantial head turns). The position of the loudspeaker (left or right of subject) was counterbalanced across subjects during test phases to limit laterality bias. During all test trials, two experimenters were required: Experimenter 1 activated the playback of each trial while standing directly behind the subject to avoid providing any cues to the subject, while Experimenter 2 stood behind the camera while looking toward the floor for the same reason (see figure 2). Each test trial was started only when the subject was (i) stationary, (ii) within frame of the camera, (iii) facing either the camera or at least 90° away from the loudspeaker and (iv) at least 15 s had passed since the last trial. To ensure the rest of the group remained naive to generalization and violation sequences until tested, we activated ventilators in the other areas of the facility (a usual procedure for the bonobos) and ensured that it masked any experimental stimuli. To do this, Experimenter 1 played familiar sequences in the greenhouse, while Experimenter 2 checked that it was not audible in the other areas of the facility.

### 2.5. Apparatus

All stimuli were played through an iPhone 13 Pro connected wirelessly to an Anchor AN-Mini series loudspeaker using Sennheiser SK 100 G4 transmitters. All familiarization and test sessions were recorded using Sony HDR-CX240E digital video cameras.

### 2.6. Quantification and statistical analysis

#### 2.6.1. Video coding

All videos were coded frame by frame using BORIS behavioural observation software (v. 6.2.2 available at https://www.boris.unito.it/) by M.L. Following the procedure of Watson *et al*. [9], we used a 15 s response window beginning from the onset of the final element in each test sequence. During this response window, the coder placed a time stamp on frames in which the subject oriented their head directly toward the loudspeaker (<45° angle between head orientation and loudspeaker) and a second time stamp when their head was no longer oriented toward the loudspeaker (>45° angle between head orientation and loudspeaker). As our response measures for analysis, we examined the total additive duration of each looking bout occurring within the 15 s response window, and the latency at which the first looking bout occurred.

#### 2.6.2. Statistical analysis

We analysed our data using Bayesian Cox proportional hazards models [17], a form of survival analysis, to examine the differences in latency and time spent looking toward the loudspeaker in the 15 s response window following each test sequence (familiar, generalization and violation). One ‘Full’ model and one ‘Null’ model were fitted for each condition (AD and Non-AD) and each outcome variable (gaze duration and response latency), making a total of eight models. Each Full model included a fixed effect of sequence type (three-level factor: familiar, generalization and violation sequences) and a random slope for individual identity to control for repeated measures, whereas the Null model contained no fixed effects and a random intercept for individual identity. For each analysis, the Full and Null models were compared to determine which had the best out-of-sample predictive fit using the difference in generic (expected) log-predictive density (ELPD) between models. Where there was no clear best-fitting model (i.e. the difference in ELPD was less than twice the standard error of the difference in ELPD between the models), the most parsimonious model (i.e. Null model) was adopted.

All models were implemented in R [18] and RStudio [19] using the package ‘brms’ [20]. All models were run with two chains, each comprised of 5000 iterations. Model chain convergence was assessed by inspecting trace plots, rhat values (all equal to 1.00) and effective sample sizes (all over 1000). All data and code used for analysis can be accessed at the following repository: https://osf.io/cv84f/.

## 3. Results

### 3.1. Adjacent dependency processing

**Figure 3.**
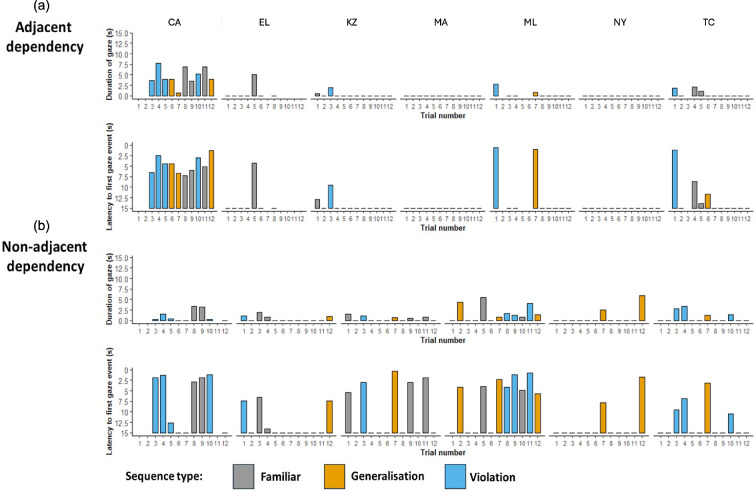
Summary of raw data for each individual (columns), condition (a: AD; b: Non-AD), and response measure (top: duration of gaze; bottom: latency to first gaze) for familiar (grey), generalization (orange) and violation (blue) sequences.

Within the adjacent dependency condition, we found no evidence that our subjects processed adjacent dependencies (figure 3). Specifically, when examining gaze duration as a response measure, neither the Full nor Null model provided a reliably superior out-of-sample predictive fit, and we therefore adopt the Null model as the most parsimonious. Meanwhile, when examining response latency, the Null model explicitly provided a better out-of-sample predictive fit than the Full model (table 1). Furthermore, even within the rejected Full models, there was no evidence of differences between any of the playback conditions (familiar, generalization and violation sequences) for either response measure (electronic supplementary material, table S2). Therefore, it is reasonable to infer that sequence type was not a useful predictor of either behavioural response.

**Table 1.** Model comparison table for each condition and response measure. Models in descending order of out-of-sample predictive power as determined by the difference in generic (expected) log-predictive density (ELPD) between models. A difference in ELPD of greater than two times the standard error of the difference in ELPD would suggest the model provides a substantially better predictive fit than the competing model. In the absence of this, the Null model is adopted as the most parsimonious. Coefficients for Full models can be found in electronic supplementary material, table S2.

condition	response measure	model	ELPD difference	s.e. of ELPD difference
adjacent dependency	gaze duration	null	0	0
		full	−0.6	0.9
	response latency	null	0	0
		full	−2.6	0.2
non-adjacent dependency	gaze duration	null	0	0
		full	−1.1	0.6
	response latency	null	0	0
		full	−2.9	0.3

### 3.2. Non-adjacent dependency processing

We found no evidence that our subjects processed non-adjacent dependencies in our artificial grammars (figure 3). As with the adjacent dependency condition, when examining gaze duration as a response measure, we found that neither model provided a substantially better predictive fit, whereas when examining response latency, we found that the Null model is superior (table 1). However, even within the rejected Full models, we found no indication that responses reliably differed between playback sequences for either response measure (electronic supplementary material, table S2). We therefore infer that sequence type (familiar, generalization and violation) was not a useful predictor of behavioural response.

## 4. Discussion

To determine whether recent data demonstrating AD and Non-AD processing in chimpanzees [9] are indicative of an ancestral origin in *hominini* of this key feature of language, we replicated this experiment with captive bonobos. In both AD and Non-AD conditions, our subjects showed no behavioural difference in their response to different playback sequences, regardless of whether they were entirely consistent with the habituation stimuli (familiar sequences), comprised of novel sound variants but structurally similar to habituation sequences (generalization sequences), or both acoustically and structurally novel (violation sequences). Therefore, we found no evidence that bonobos share the ability to spontaneously process ADs and Non-ADs in novel acoustic sequences with humans and chimpanzees.

What might explain the apparent inability of bonobos to apply dependency processing to novel acoustic structures? Like chimpanzees [21–23], bonobos naturally combine multiple calls from their vocal repertoire into larger structural configurations that also appear to be meaningful to the signaller [24–27]. Hence, our findings cannot be attributed to species differences in combinatorial communication more generally. One possibility is that, unlike chimpanzees, bonobos do not readily generalize these combinatorial abilities to novel sequential structures. This hypothesis is perhaps supported by the fact that chimpanzee and bonobo vocal systems have diverged rapidly since their common ancestor [28]. This arguably entails parallel divergences in certain aspects of their perceptual systems, which may be pertinent to the processing of artificial grammar. Interestingly, a comparable inability to process novel, artificial grammars is mirrored by our previous work in chestnut crowned babblers [10], a species of cooperatively breeding songbirds. Like bonobos, these birds produce complex vocal structures. In babblers, these structures could even be characterized as containing non-adjacent dependencies [29], but they were nevertheless unable to process the Non-AD condition when this experimental approach was applied to them.

Whilst our data do not support an ability for bonobos to spontaneously process Non-ADs in artificial grammars, Non-AD processing is not beyond the absolute capabilities of this species. One of our subjects was the language-trained ape ‘Kanzi’, who has previously demonstrated considerable language-like competence, including an ability to comprehend complex novel sentences spoken in English (e.g. [30]). Given Kanzi’s clear ability to process both ADs and Non-ADs, it is even more challenging to explain why he did not show a pattern of results consistent with dependency processing in the present study (see subject ‘KZ’ in figure 3). One explanation might be that Kanzi’s processing of linguistic syntax is scaffolded by his processing of semantics, whereby the relations between elements in a structure are easier to process if those elements themselves bear meaning (as opposed to our entirely abstract, artificial sequences) [3]. Alternatively, the extensive exposure of Kanzi and the other subjects to behavioural experiments and human-introduced activities may have confounded their response to our entirely passive experimental design. For instance, relative to typical bonobos, these frequently tested subjects may be less surprised by novelty in their environment or simply less motivated to immediately investigate it [13]. This factor may have been compounded by the fact that unlike Watson *et al*. [9], due to sample availability, we tested the same individuals in both AD and Non-AD conditions (albeit with a seven month break). As a result, subjects may have demonstrated muted responses to our passive playback design, even if they possessed the capacity to process our artificial grammars, as is sometimes the case in adult humans [31,32]. This explanation may be further supported by the high individual variation observed within our subjects: individuals that have been tested the least intensively (e.g. individual CA arrived only 3 years before testing) also happened to be the most responsive subjects in the context of the current experiment (figure 3). Unfortunately, our sample size was not large enough to statistically explore this potential confound. Another point of divergence between the present study and Watson *et al*. [9] is that, within a condition, the bonobos were all exposed to the same artificial grammar, whereas each half of the chimpanzee sample was exposed to different forms (e.g. Individual 1 learns a grammar where A–B and C–D are dependent elements, while Individual 2 learns A–D and C–B) to control for potential differences in learnability between different sound elements. Although Watson *et al*. [9] did not find that the behaviour of subjects differed depending on which grammar they had been habituated to, and all bonobos were exposed to the theoretically most easily learned combinations of elements (where dependent elements were most similar to one another), it is possible that the grammar of dissimilar dependent elements would somehow be more readily learned by bonobos.

There are several possible ways of revising our methods to overcome the limitations of passive designs. For instance, active tasks have been used in both adult humans [30] and non-human species to rule out motivational confounds (e.g. a go/no-go procedure, as used to test for Non-AD processing in various species [33]). However, such approaches typically take enormous amounts of training when applied to non-human species, constraining feasible sample sizes and arguably diminishing ecological validity of the test [5]. Alternatively, the passive learning design deployed in the present study could be augmented via biometric technology, which can probe subjects for more subtle physiological responses to stimuli rather than overt behavioural change. For instance, thermographic cameras are a powerful method of measuring changes in arousal states in response to acoustic stimuli in apes [34] and monkeys [35] and could be incorporated into the present experimental approach.

Despite these methodological concerns, taken at face value, these findings potentially complicate previous arguments that dependency processing was extant in our last common ancestor with *Pan* [9]. To disentangle the most plausible evolutionary scenario for Non-AD processing in human and non-human primates (i.e. convergent evolution versus homologous traits), necessary data from gorillas and orangutans are still missing. An inability to process Non-ADs in gorillas and orangutans would support a convergent evolutionary scenario for this trait, where the capacities observed in chimpanzees and marmosets [9] evolved independently in their unique lineages. Conversely, data confirming Non-AD processing in these other ape species would support a common descent evolutionary scenario, with the absence of Non-AD processing in bonobos perhaps attributable to a disappearance of the ability in this branch.

In conclusion, our artificial grammar learning experiment provides no evidence that bonobos process adjacent or non-adjacent dependencies in novel sequential acoustic stimuli. This presents a striking contrast with directly comparable experiments demonstrating that chimpanzees and common marmosets were both able to process these syntax-like structures [9]. These findings might suggest a convergent rather than ancestral evolutionary origin for this capacity. However, the extent to which these differences can be attributed to a genuine inability of bonobos to process adjacent or non-adjacent dependencies is complicated by the extensive research history of our sample. Further work with additional populations of bonobos, as well as other species of great ape, will shed further light on when this key feature of language originated.

## Data Availability

All data and code used for analysis can be accessed at the OSF repository [36]. Supplementary material is available online [37].
